# Towards elimination: Challenges in community participation to a gHAT ‘screen and treat’ strategy using the new oral drug acoziborole in the Democratic Republic of the Congo

**DOI:** 10.1371/journal.pntd.0013197

**Published:** 2025-06-20

**Authors:** Catiane Vander Kelen, Alain Mpanya, Ruth Nzuzi, Gérard Watakembi, Cathy Mbuyi, Elena Nicco, Epco Hasker

**Affiliations:** 1 Institute of Tropical Medicine, Antwerp, Belgium; 2 Programme National de la Lutte contre la Trypanosomiase Africaine, Kinshasa, Democratic Republic of Congo; 3 Freelance anthropologist, Institute of Tropical Medicine, Kinshasa, Democratic Republic of Congo; University of Liverpool, UNITED KINGDOM OF GREAT BRITAIN AND NORTHERN IRELAND

## Abstract

Until recently, treatment options for gambiense human African trypanosomiasis (gHAT) have been limited and toxic, negatively impacting community participation to screening and treatment. A new, non-toxic, single-dose oral drug has shown efficacy in a Phase III trial and is being tested in a trial called ‘STROGHAT’, which aims to demonstrate cessation of HAT transmission using a ‘screen and treat’ strategy. This study aims to explore community perceptions about current and future screening and treatment strategies and identify what could act as barriers to participation in order to prevent them. We conducted 8 focus group discussions and 18 semi-structured interviews with communities, community leaders and mobile unit managers in 4 selected villages out of 74 endemic villages included in the STROGHAT study. Our results highlight four main potential barriers: the rarity of cases has led to gHAT being perceived as a disease that no longer exists and participation to screening as a waste of time. Lack of awareness of new treatment and screening procedures perpetuates fears and misconceptions about treatment toxicity, lumbar puncture and mandatory hospitalisation. The introduction of a single-dose oral drug to be administered on the spot raised the issue of side effect monitoring and care. Finally, the lack of sensitivity to community cultural norms in the organisation of screening discourages people from participating. Those barriers are important to anticipate and include in elimination strategy. Also a monitor perception about acoziborole screen and treat during all the process through other social science based research is to foreseen.

## Author summary

Sleeping sickness is a neglected disease caused by a parasite called Trypanosoma brucei gambiense. If left untreated, sleeping sickness is fatal. Until recently, treatment has been limited and toxic, affecting community participation in screening and treatment. This has been a barrier to achieving the 2030 WHO elimination goal. Today, however, a new oral drug with no side effects is being tested: acoziborole. The use of this single-dose oral drug would simplify the process of screening and treatment, allowing a “screen and treat” strategy to be used for all suspected cases on the spot, without the need for complex confirmatory testing. For this to succeed, it will be necessary to have full community participation in active screening and treatment. Ensuring community participation can be a real challenge in the fight against sleeping sickness. A number of studies have highlighted the various barriers that prevent people from participating in active gHAT screening and treatment. We therefore undertook a qualitative with to aim to identify potential bottlenecks and encourage participation in a future screening and treatment strategy. We found that the community was receptive to the new oral sleeping sickness drugs, but had some concerns about possible side effects.

## 1. Introduction

*Gambiense* Human African trypanosomiasis (gHAT) is a neglected tropical disease caused by a parasite called trypanosoma brucei gambiense. The disease is transmitted from human to human by a vector, the tsetse fly, and is fatal if left untreated. Hundreds of thousands of deaths have been reported in the past in the Democratic Republic of Congo (DRC). Until recently, treatment options for gHAT were limited and toxic, forcing control programmes to avoid “over-treatment” by complex diagnostic procedures involving screening with serological tests followed by technically demanding and laborious microscopic confirmation of screening test seropositive subjects. If parasites were found, a lumbar puncture was required to determine the stage of the disease. This often led to patients dropping out during the diagnostic process or even not starting it at all. In addition, microscopic confirmation tests have imperfect sensitivity. Robays et al in 2004 estimated that for these reasons in active screening campaigns half of the prevalent cases of gHAT were missed [1,2]. This has important implications for control of gHAT as humans are assumed to be its main reservoir.

Until 15 years ago, treatment consisted of injections of pentamidine for stage 1 of the disease and melarsoprol for stage 2. The latter is an arsenic derivative associated with 5–10% mortality due to adverse events, particularly encephalopathy [3]. From 2009 onwards a less toxic treatment was introduced, nifurtimox-eflornithine combination therapy (NECT) [4]. Although this treatment is far less toxic, it requires intravenous perfusions and therefore hospitalisation for more than one week. Significant adverse events do still occur. In 2019, fexinidazole, a non-toxic drug in the form of oral tablets, was approved [5]. Fexinidazole may require a lumbar puncture in case of severe disease, which is preferably treated with NECT if stage 2 is confirmed. Fexinidazole is taken orally over 10 days once a day, requiring medical supervision and patients to travel to the health centre.

Recently, a non-toxic, single-dose oral drug, acoziborole, has emerged from the development pipeline and shown 98.1% efficacy in a phase III trial, irrespective of the stage of the disease [6]. Acoziborole could eliminate the need for a lumbar puncture, and could be safe enough to treat serological suspects without microscopic confirmation [7]. The ultimate goal would be to introduce a “screen and treat” strategy, in which every person with a positive screening test (either the card agglutination test [CATT] or a lateral flow rapid diagnostic test [RDT])would be presumptively treated without further confirmatory tests. A single dose of acoziborole would be administered directly on the spot.

A study called STROGHAT (Stop Transmission of gHAT) has started in April 2024 in the DRC [8]. The aim of the STROGHAT study is to check whether expanding treatment with acoziborole to all screening test seropositive subjects, including non parasitologically confirmed, for three consecutive years, can lead to a zero prevalence of gHAT in an endemic focus [9]. In order to carry out the STROGHAT study and verify its impact on transmission, it will be necessary to have a full community participation to active screening and treatment. Ensuring community participation can be a real challenge in tackling gHAT. A number of studies have already highlighted the various barriers that dissuade people from taking part in active gHAT screening and treatment, such as fear of lumbar puncture, blood sampling, adverse events of treatment, lack of confidentiality or waste of time. [1,10–16]

The STROGHAT study is carried out in a gHAT endemic focus in the Equateur Nord region, in the north-west of the DRC. To date, no study on the factors influencing participation in screening and treatment has been carried out in this region. Although epidemiological data show a good level of participation in active screening in this region this does not always mean that acceptability can be taken for granted. We therefore carried out a qualitative study of the population’s perception of screening and treatment to further optimise participation and identify bottle necks. The study objective was to identify possible bottle neck and leverage participation in a future screen and treat strategy. To do this the study first explored perceptions of the existing strategy based on active screening, confirmation and treatment. Secondly, it aimed to understand the community’s perception on future single-dose oral treatments in the context of gHAT.

## 2. Methods

### Ethical approval

The research protocol was approved by the Institutional Review Board of the Institute of Tropical Medicine, Antwerp, Belgium (ref: 1702/23) and the Ethics Committee of the Protestant University of Kinshasa (ref: CEUPC 0110). Informed consent for the study was obtained from the authorities of the province of Equateur Nord and the village chiefs prior to data collection. All participants were informed of the research objectives, their voluntary participation and their right to withdraw from the study. Verbal consent was audio recorded, to allow analphabetic people to feel comfortable.

### 2.1. Qualitative study sites and population

**Fig 1:**
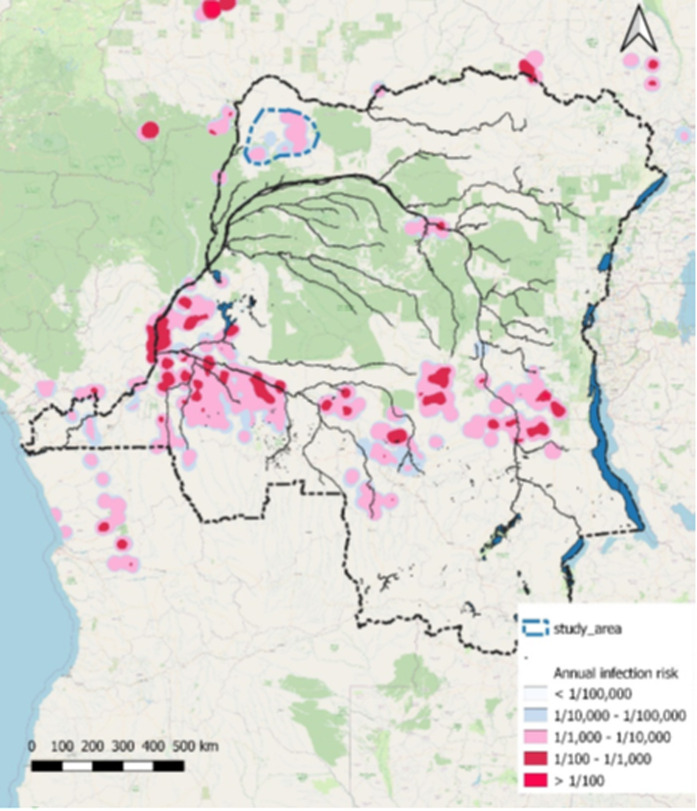
Selected area in Equateur Nord, blue (Original work Elena Nicco, shapefile source: OpenStreetMap https://www.openstreetmap.org/#map=7/2.362/20.709).

**Fig 2:**
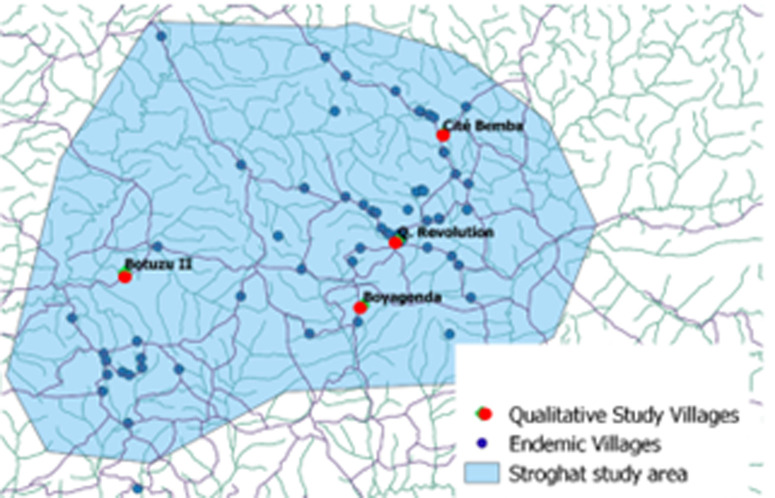
The 4 villages of the qualitative study (Original work Catiane Vander Kelen, shapefile source: OpenStreetMap https://www.openstreetmap.org/#map=7/2.362/20.709).

The STROGHAT study will take place from 2024 until 2027 in Equateur Nord, DRC (Fig 1). The study area was selected on the basis of endemicity and accessibility criteria and because it is surrounded by gHAT non-endemic areas. It comprises of 403 villages, 74 of which are considered endemic and will be screened on an annual basis by three mobile units (MU) and one mini mobile team. For the qualitative study, we chose 4 villages from these 74 (see Figs 1 and 2). These villages were chosen to represent as much diversity as possible (different health zones, low and high participation rates, distances from referral hospital) (see Table 1).

**Table 1:** Qualitative study village characteristics.

Village name	Health Zone	Number of cases of gHAT (5 years)	Nearest Hospital	Distance to nearest hospital	Screening participation rate
Boyagonda	Bominenge	1	Karawa	17 km	43%
Q. Revolution	Karawa	1	Karawa	5 Km	94%
Cité Bemba	Loko	1	Loko	50 Km	96%
Botuzu 2	Bwamanda	1	Gemena	6 Km	92%

The population of Equateur Nord is predominantly rural. The main livelihoods are agriculture, fishing and livestock. The main official language is Lingala, but the main local language is Ngbaka. The region is very rich in its diversity of languages, ethnic groups and religions. It is home to the Ngbaka, Bangala, Mbanza, Mono, Ngombe and Ngbandi ethnic groups, to name only those identified in the villages included in this study. Several religions are also practised: Catholic, Protestant, Kimbanguist, Muslim, Nzambe Malamu or “assemblée des frères”. These are patriarchal communities and most positions of power are held by men, although women were found to be clan chiefs in some of the villages studied. In general, participation in active screening for gHAT is good, and the population shows respect and confidence in local authorities and national institutions.

### 2.2. Data collection method

Two qualitative methods were used to collect data: Focus Group Discussions (FGDs) and Semi-structured Interviews (SSIs). Two FGDs were conducted per village (one with men and one with women). A total of eight FGDs were conducted, four with women (aged 30–51), three with middle-aged men (aged 35–55) and one with young adult men (aged 21–29). A group of young adult women was also planned, but this was not possible due to a misunderstanding with the data collectors. A total of 18 SSIs were conducted, 16 with community leaders (4 per village) and 2 with PNLTHA chiefs of MU, as they have a general vision of the MU challenges. Community leaders are people who are recognised as having a mobilising role and influence on particular groups in the community, such as village chiefs, community health workers called ‘relais communautaires’ or ‘RECOs’, religious leaders or association presidents. Of the 16 SSIs with community leaders, there were 4 village chiefs SSIs, 10 RECOs, 1 youth president, 1 member of a health area development committee (CODESA). Unfortunately, it was not possible to identify any female leaders to participate in this study except one female RECO. Female generally do not held power position. This data was collected in October 2023.

The SSIs and FGDs took place in private places, such as churches or classrooms, chosen by the participants at their most convenient time. The SSIs lasted an average of 35 minutes and the FGDs an average of 1 hour. All were conducted in Lingala by two experienced anthropologists (RN and GW). All SSIs and FGDs were audio-recorded with the permission of the participants. A topic guide was used as support for the FGDs and SSIs to cover the themes of current perceptions of the screening and treatment and future use of acoziborole.

### 2.3. Data analysis

The audio-recorded FGDs and SSI were translated from Lingala into French and transcribed into a Word document. The translation was checked by members of the research team who were fluent in French and Lingala (RN and GW). All the transcripts were then analysed by the three anthropologists in the team using thematic analysis. This method combines a deductive approach, in which the data are analysed according to themes predefined in the interview guides, followed by an inductive approach, in which new themes are identified. All transcripts were carefully read and reread to recognise and identify patterns and to compare similarities and differences in the participants’ discourse. NVivo software (V.11; QSR International, Melbourne, Australia) was used for data analysis.

## 3. Results

The results of the study are presented in two parts. The first part reports on the current perceptions of screening and treatment and identifies the factors currently influencing people’s participation. This part has three sub-themes. The second part reports on the perception of a future single-dose oral tablet for the treatment of gHAT and identifies possible additional influencing factors. This section has three sub-themes. There are no notable differences in the discourse between the men’s and women’s FGDs, although the young men’s FGDs shows some particularities. The mobile unit managers and leaders express perceptions similar to those identified in the FGDs and they shed light on the relational aspect between the MUs and the community.

### 3.1. Factors influencing participation in screening and treatment of sleeping sickness in the Equateur North region.

#### 3.1.1. “Dangui” (sleeping sickness) is perceived as a serious disease but very rare and of the past.

“Dangui” means sleeping sickness in the local Ngbaka language. Participants perceive it as a serious illness because it has physical and social consequences. It is known as fatal and is associated with serious symptoms such as madness, behavioural disorders and impotence. It is a source of family conflict and rejection because it is associated with evil spirits and witchcraft. Nevertheless, the participants associate sleeping sickness with the past, more specifically with the colonial era, and today they perceived it as fairly rare. They therefore feel less concerned, especially young people.

*“Sleeping sickness is really very dangerous, it killed a lot of people in the 60s, before independence, the Belgians were still in the Congo, Maman! Now this disease is rare, we’re not going to find any more cases here.” (FGD, young adult man, Cité Bemba, October 2023)*

In consequence, screening is seen as a waste of time interfering with work in the fields because the participants feel discouraged by results that are very often negative. The rarity of the disease provokes frustration and even creates suspicion. The fact that people come to screening sometimes feeling sick, but are not diagnosed make them feel their results are being kept from them. Some people wonder whether this screening might not have a hidden purpose.

*“They take your blood for tests, but they don’t give you the results, they just tell us ‘it’s negative’, but they stay with the blood there and people say that our blood is taken to Europe for I don’t know what, that’s why they’re now afraid to come for screening.” (FGD, young men, Cité Bemba, October 2023)*

#### 3.1.2. Despite fexinidazole oral treatment and simplified screening and care, fear of the side effects, lumbar puncture, toxic treatment and a perceived high indirect cost are still present in people’s minds.

Although lumbar puncture and NECT are currently only required for very advanced cases and the use of fexinidazole tablets is preferred, this does not seem to be known by the population. Screening is still strongly associated with lumbar puncture and its side-effects, and treatment is still associated with toxic and potentially lethal products.

Although outpatient treatment in health centres could now be possible, people still associate it with hospitalisation. Even if they are aware that the treatment is free, hospitalisation represents a significant cost for patients and their families: transport to the hospital, food and the cost of a family member’s absence from the field for 10 days.

*“They give you the treatment for free, but when you go there it’s very far away and no one will give you any food there, because you are alone. But you know that the medicine causes reactions if you don’t eat properly and you can die. That’s why, because we don’t have the means, we prefer to avoid treatment if we’re ill... it’s better to die with the illness than with the side-effects of the treatment and hunger. So some people say, why even go for screening if I can’t afford it? (ESS, RECO, male, Boyagonda, October 2023)*

Regarding fexinidazole and related new care procedures, participants report that there has been no awareness of gHAT and related care for a very long time.

*“We only pass on the message that the mobile team has come for the screening and not to move, to come to the screening, that the screening is free and that the screening will take place in the village chief’s home under a tree or in church, for example. Apart from that, there’s no other information. (FGD, Botuzu men, October 2023)*

#### 3.1.3. Mobile Unit screening organisation has improved however some practices remain as barriers: lack of privacy, community cultural norms and community rhythm.

Screening is mainly taking place outdoor in public. Sleeping sickness is associated with witchcraft and is therefore highly stigmatised, with potential social consequences. Witchcraft suggests that the patient and/or his family are responsible for the illness. The fact that screening is taking place in the open air reinforces the fear of being rejected by peers.

*“If you’re caught with sleeping sickness, you become a subject of mockery in the village, people will start saying that you’re bewitched, they won’t come near you, because you’re dangerous” (FGD, woman, Botuzu, October 2023).*

The way in which screening is organised is also an issue in relation to other cultural norms in the community. Participants describe how there is a sense of shame in showing that you are in pain, especially for men in front of their wives and children. They also describe how the queue does not respect the priority of elders who should not wait standing after their children.

*“I don’t think it’s right to do the tests out in the open like that, queuing as pupil, this is humiliating to see ancient behind me. I don’t take part in the screening either, because they catch you and you become a laughing stock, they prick you and you scream in front of your children and your poverty is going to get out, I’d rather die, Maman” (FGD, men, Boyagonda, October 2023).*

Mobile Unit chiefs report in their interviews that adapting the screening schedule to the community’s daily rhythm (early in the morning before field work and late in the afternoon) improved community participation. However, community members point out that at certain times of the year, part of the population goes to work in the forest for several weeks or even several months during the semi-sowing period (September-October) and the harvesting period (which varies depending on the crop). As a result, they are unlikely to be counted in the pre-screening census, they are not screened because they are not informed.

*“People goes into the forest to work in the fields for long period, and many miss the screening because they don’t even know when it is. They go to their camps in the forest for several weeks. The team needs to be able to go there and screen them” (ESS, RECO, woman, October 2023).*

On the other hand, participants also identify some levers that positively influence participation in the screening organisation. They mention the importance of involving chiefs and some trusted people. For example, one RECO mention in an interview that people often go because they respect and trust their village chiefs, who asked them to go, rather than because of any collective effort to eradicate the disease.

*“There are people who sacrifice their time to come to the screening, but we come for nothing and we stay in line like little children. We only go because the capita (village chief) asks everyone to go”. (ESS, Recos, male, Boyagonda, October 2023)*

In this context, the involvement of trusted people such as the chiefs, the RECOS, the “Infirmier Titulaire” (nurse in charge of health centre) is essential. The Mobile Unit chiefs underline this important aspect for the success of the screening and insist that the RECOs and chiefs should be motivated (financially). The chiefs and RECOs who accompany the work of the MUs have a real loss of income if they do not go to the fields during the screening period. If no compensation is introduced, the MU chiefs fear that their good relations and willingness of community leaders to support the MUs will be damaged. This feeling could be exacerbated in a context where cases of disease are rare.

*“A village can hold 5,000 people, so the RECOs and the chiefs have to stay until everyone has passed through. This makes it very difficult for them to deal with the problems in the fields. The RECOs are used to distributing mosquito nets, vaccines and receiving a small payment. But we haven’t planned for that. That’s why we can arrive in a village and they’re all off in the fields, and that delays the work. But this little thing could improve screening” (ESS, MU Chief, October 2023).*

### 3.2. The perception of a single-dose tablet for gHAT

#### 3.2.1. Perception of oral medicines to cure serious diseases.

Participants in the study describe 3 types of medication to treat illnesses: plants, tablets and injections. Plants are available locally and are free, so they are the first choice. If that doesn’t work, people turn to tablets (such as paracetamol) bought from pharmacies. Plants and tablets bought from pharmacies are associated with “minor ailments” such as fever, headache and even malaria. They are associated with self-medication or traditional medicine and are taken locally.

*“When we are ill, there are many plants in the forest that heal or we go to the chemist to find the remedy. You can ask another person who has had the same experience. For example, someone else may tell you that when my child was ill, I gave him this medicine, and as your child has almost the same symptoms, you can try the same medicines. When you go to the health centre, you have to have money” (FGD, men, Boyagonda, October 2023).*

There are tablets that are prescribed and therefore administered by professionals in health facilities. These tablets are perceived to be for potentially serious conditions and to have more side effects. However, they are also perceived as slower to respond than injections because they have to be taken over a longer period of time and tend to weaken the person. For serious illnesses, many participants say they would prefer injections because of their rapid onset of action and therefore perceived shorter treatment. Injections are for serious conditions and are associated with health facilities, particularly hospitals. In fact, for some people, receiving good treatment in hospital is equivalent to receiving injections. Injections are perceived to be more expensive, faster-acting and stronger, but they are also perceived as more risky because they have more side effects. Table 2 summarises the perceptions of plants, tablets and injections.

**Table 2:** Summary of the perception of medicines identified in the data.

	Not serious illnesses		Serious illnesses	
symptoms	Fever, headaches		High fever, severe diarrhoea, vomiting, unable to eat	
Medicines	Plants	Available off the shelf tablets	Prescribed tablets	Injections
Place of administration	Village	village	Health centre, then village	Health structure (mainly hospital)
Cost	Free 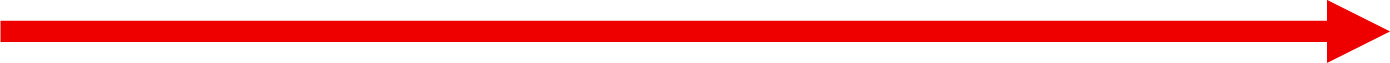 Expensive			
Speed/ efficiency	Slow 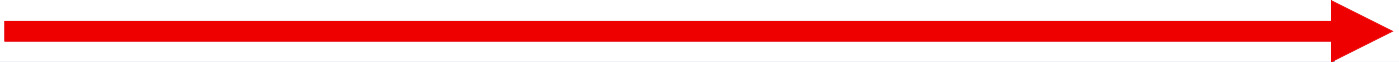 Fast			
Risks of side effects	Low 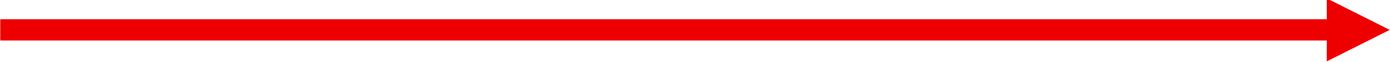 High

*“Injections are very risky, my daughter is now ten years old, in 2021 we admitted her to hospital and treated her with injections because her condition was serious, but after the treatment we noticed that her foot was swelling where she had been injected and this created an abscess and to this day my daughter has a deformity as a result of the injections” (FGD, women, Botuzu, October 2023).*

#### 3.2.2. Perception of gHAT tablets: “Reducing the distance between the community and the treatment! It will save lives, it will be fantastic!.

Participants unanimously welcome the prospect of a future single-dose oral tablet for the treatment of gHAT. This, of course, echoes the issue of indirect costs associated with treatment and care.

*“ Medicines will really help a lot of people. We’re poor here and to get treatment you have to travel a long way, pay for transport and accommodation, it’s expensive. Some people can’t afford it and die. But if the medicines are brought to us, it will be a good thing for the community” (FGD, women, Botuzu, October 2023).*

Many participants immediately associate taking oral tablets as a new treatment with the cessation of lumbar puncture.

*“It’s a good thing, people used to run away from injections if we get this medicine it will be a really good thing and I don’t think people will run away from treatment any more, because I was used to be scared of injections, especially the one in the spine” (FGD, Botuzu woman, October 2023).*

If there is good awareness, participants are confident that acoziborole should be well accepted by the community.

*“Even if they are used to medicines, they may find it strange that there is a pill for trypanosomiasis, but they will accept it without any problems unless there are side effects”. (ESS, RECOS, male, Quartier Révolution, October 2023)*

#### 3.2.3. Perception of gHAT tablets: Concern about taking a tablet administered outside a health facility.

Despite their enthusiasm, participants highlight certain elements that could potentially hinder their participation. They compare taking a single-dose tablet for gHAT with their experience of vaccination campaigns or the door-to-door distribution of ivermectine for lymphatic filariasis in villages. Although acoziborole will initially be distributed in health facilities as part of the STROGHAT trial, the comparison provides some interesting elements for the future.

a *Managing side effects and overdose*

For the participants, taking medicines in the community, outside the structures, is not without risk. Medicines that are not available off the shelf and therefore prescribed are stronger and are given within the framework of a structure that can ensure follow-up in the event of problems. The medicines distributed in the village are administered by health staff and are not off the shelf. They are associated with potentially serious side-effects and are perceived as being unsupervised.

Participants emphasize that the main problem with these free drug distributions carried out by organisations independent of the health centre is that there is no **follow-up** regarding side effects. As a result, taking the medicine can be perceived as causing illness or even death, when the person was feeling perfectly well in the first place. To deal with this, they suggest leaving free medicines to treat any side-effects at health posts or health centres, and they also suggest getting these facilities involved so that they can provide free follow-up.

*“Very often the medicine we’re given for free creates illnesses after the organisation goes. When you go to the health centre for your side effects, they ask you for money and if you don’t have the money you can lose your child just like that. Which is why organisation needs to work with our health posts and health centres. (FGD woman, Cité Bemba, October 2023)*

**Overdosing** was also one of the concerns raised by participants, many of whom made the link between serious side effects and overdosing, especially if the person had not eaten properly beforehand. They were also concerned about tablet distributors calculating the correct dose according to the person’s weight, age and height, and feeding people beforehand.

*“You have to take into account the dose, you have to take the person’s height and weight and also know if the person is eating well, here we don’t eat well and if you come with medicines that are very strong, it’s going to kill us again.” (FGD, women, Botuzu, October 2023)*

b *(no) Confidence in the organisation administering the medication*

A problem of **trust** is also mentioned in relation to the free distribution of medicines. Some mention rumours that these drugs would have another purpose, such as killing people. Organisations distributing ivermectine or doing vaccinations are often vertical programmes and are perceived by people as being outside the health system and sometimes even linked to “white people” and this seems to shake people’s confidence in the products.

*“Our children suffer a lot from diarrhoea and others die, but when we lose children like that, without knowing the causes, other people say it’s because of white medicines that are distributed free of charge. I know that you (the interviewer) have our black skin, so you can’t hurt us like the white man. But because of him, we refuse”. (FGD, women, Botuzu, October 2023)*

The person who gives the medicine and is responsible for distribution is therefore of vital importance, and the participants are very insistent that people in the community or trusted health structures should be involved.

*We don’t want you to be able to send us someone from outside for fear that nothing good will happen to us, which is why we want ourselves, the chief and the RECOs to be able to manage this (FGD, men, Boyagonda, October 2023).*

Concerns about serious side-effects, overdosing and the involvement of “white people” are probably of particular importance when it comes to gHAT, which is associated in the collective consciousness with treatments dating back to the colonial era, to toxic treatment that potential lead to death.

*“You have to follow up each person who drinks this, because the medicines we used to give led too many people dying, instead of the person recovering, the medicines killed them, so you have to follow up after three months to see the side effects”. (FGD, men, Botuzu, October 2023)*

## Discussion

This study shows that there are still many real or perceived community concerns about the current and future screening and treatment strategy of gHAT that hinder community participation and create obstacles to gHAT elimination.

The main obstacle is, paradoxically, the epidemiological situation with very few reported cases of gHAT. This new reality shapes the perception that the diseases are no longer a threat. This phenomenon was also reported in the most recent study on the perception of gHAT in the DRC, conducted in the Kasai and Bandundu regions in 2022 [11]. The decision to participate in a health control programme is generally based on a balance between potential benefits (relief of suffering, reduction of stigma and financial burden of the disease) and potential harms (side effects of medication, time and cost impact on livelihood) [17–24]. Although, with a future “screen and treat” strategy using acoziborole the screening and treatment will be significantly simplified, the rarity of a disease still may negatively influence the decision to take part in disease control activities, as observed by different authors on mass drugs administration (MDA) in the NTD field [19,21,25,26]. MDA is generally a straightforward strategy that takes a limited time and is free, however authors observe that some people refuse to take the drugs because they do not see the benefit compared to other perceived more important community health problems [21,26]. The perception of low benefit in taking part to control activities for a disease with low prevalence can also be amplified by local perception of pharmacology [18–23,27]. A study in Tanzania on trachoma antibiotic MDA put in evidence that people’s participation is challenged by the perception that injections are much more effective than oral treatment [21,28]. Acoziborole will be given based on a “screen and treat” strategy, therefore only screening test seropositive subjects will be treated and this is probably an advantage allaying this concern.

Regarding the use of a single-dose oral medicine to treat gHAT, community participants highlight some benefits, such as reducing distance, time constraints and indirect costs. However, they also express strong concerns about potential harms due to potential side effects and lack of follow-up. Our study participants clearly mention, based on previous experiences with anti-lymphatic filariasis (LF) drugs, that taking the drug could potentially cause illness or even death when the person was feeling perfectly well in the first place, and that there is no follow-up period after the drug intake because of vertical interventions. Kisoka’s study of LF shows the same perception [21]. People interviewed in this study insisted that they should be screened first, rather than given mass treatment to everyone, to limit harms.

Participants emphasize the lack of awareness and information about gHAT, and as a result they still think old procedures are applied, such as toxic treatment, lumbar puncture or mandatory hospitalisation. There is a lack of information about the simplification of gHAT care. This lack of knowledge about new tools and strategies is also reported in studies on the perception of gHAT in the DRC [2,10,11]. Our study highlights and confirms that awareness of the new oral treatment, fexinidazole, is also low in the Equateur North region.

Finally, different studies as well as our study participants, highlight the importance of the process [19–23,27,29]. The perception of the programme and the decision of taking drugs is influenced by where, how and especially by who the drugs are administered. A study in Kenya on LF shows, for instance, that poor communication and distrust of the federal government and the poor knowledge, support and supervision of drugs distributors, represent an obstacle to participation [19]. The participants of our study mention the importance of involving people whom they trust and to exclude people whom they do not trust. In one hand, vertical programmes, such as ivermectine distribution for LF are perceived by the participants of the study as being outside the health system and often linked to “white people” and this seems to inspire suspicions based on historical colonial backgrounds. On the other hand they seem to trust local leaders such as the village chiefs, health centres nurses (Infirmier Titulaire, IT), RECOs and local health and administrative authorities, to administer the drugs and also to follow-up on side effects after the PNLTHA leave their village. In addition to “who” is administrating, the “how” it is administered is also very relevant. As also reported in other study on gHAT perception, they particularly recommend and insist on more privacy to avoid stigmatisation and its social consequences [2,10,11]. For that matter some participants mention they would prefer the drug to be administrated at the health centre instead of the village. As a study in Sierra Leone suggests it is better to have several administration points, to suit people’s preferences which may be diverse [22].

Based on our study, four main recommendations can be made to improve participation in acoziborole “screen and treat” activities in Equateur Nord and later elsewhere. Although some of them are already anticipated in the strategy proposed by STROGHAT, attention needs to be paid to their actual implementation. Firstly, good awareness raising, continuous communication and more qualitative acceptance and perception studies are urgently needed and need to be seriously invested in. This is important to ensure maximum understanding of the benefits of acoziborole and to limit suspicions, rumours and concerns. Although awareness is essential, it is far from sufficient. Too many programmes invest only in awareness campaigns, hoping that information will automatically increase participation. It is therefore also important to question the way in which programmes implement their activities [30]. This brings us to the second recommendation, a huge effort needs to be made on privacy and respect for cultural norms during screening. The community needs to be involved and consulted and their input needs to be encouraged as much as possible. Trusted people in the community need to be identified, specifically involved and financially motivated. Thirdly, ensuring good follow-up of side effects is probably the key point for acceptance in drug administration. This is planned in the STROGHAT study. Finally, the last and probably most challenging recommendation is to regain the interest of the population in gHAT in this low-prevalence context. For this, some participatory research needs to be done to see how we can include gHAT activities in a wider context that would meet the more important perceived needs of the community.

## Limitations

The scope of our study is obviously geographically limited, even if it is enough to draw useful conclusions for the main intervention trial. Gender perspectives and power relations could have been emphasized more in order to have a deeper understanding in decisions related to participation in a screen and treat program. Also an interviewer bias may have been introduced with the MU chiefs, as data collectors were linked to the PNLTHA coordination in Kinshasa.

## Conclusion

This paper explores the potential barriers to community participation in a future screen and treat strategy in order to anticipate bottlenecks as far as possible. Although acoziborole treatment and its screen and treat administration strategy will greatly simplify gHAT control, some barriers are identified. The current epidemiological situation of low prevalence discourages people from participating. Lack of awareness of new treatment and screening procedures perpetuates fears of treatment toxicity, lumbar puncture and the significant indirect costs of hospitalisation. The introduction of a single oral drug for gHAT raises the issue of potential side effects and their management. Finally, recommendations are made to better consider cultural norms when organising screening and to involve trusted community members. For a successful screen and treat strategy, actions need to be taken to reduce those obstacles. Follow up qualitative studies have also to be foreseen to identify remaining and new obstacles while the screen and treat strategy is implemented.
