# Correction: Towards elimination: Challenges in community participation to a gHAT ‘screen and treat’ strategy using the new oral drug acoziborole in the Democratic Republic of the Congo

**DOI:** 10.1371/journal.pntd.0013692

**Published:** 2025-11-10

**Authors:** The PLOS Neglected Tropical Diseases Staff

The initials of the first author are indexed incorrectly in PubMed. The correct initials are, respectively: Vander Kelen C.

The correct citation is: Vander Kelen C, Mpanya A, Nzuzi R, Watakembi G, Mbuyi C, Nicco E, et al. (2025) Towards elimination: Challenges in community participation to a gHAT ‘screen and treat’ strategy using the new oral drug acoziborole in the Democratic Republic of the Congo. PLoS Negl Trop Dis 19(6): e0013197. https://doi.org/10.1371/journal.pntd.0013197.
